# Prevalence and predictors of implanon utilization among women of reproductive age group in Tigray Region, Northern Ethiopia

**DOI:** 10.1186/s12978-017-0320-7

**Published:** 2017-05-18

**Authors:** Desta Gebre-Egziabher, Araya Abrha Medhanyie, Mussie Alemayehu, Fisaha Haile Tesfay

**Affiliations:** 1 Integrated Family Health Program (IFHP), Tigray Branch, Mekelle, Ethiopia; 2 School of Public Health, Mekelle University, College of Health Sciences, Mekelle, Ethiopia

**Keywords:** Implanon, Long acting contraceptive methods, Contraceptives, Implants, Ethiopia, Family planning, Health extension workers, Community based interventions

## Abstract

**Background:**

The Ethiopian Federal Ministry of Health introduced provision of the contraceptive Implanon at community level by trained health extension workers in 2009. However, little is known regarding the utilization and factors associated with Implanon use among rural women since the introduction of the community based intervention. Thus, this study assessed the utilization of Implanon and associated factors among reproductive aged women in rural areas of Saesie-Tsaeda Emba and Ofla districts in Tigray, Northern Ethiopia.

**Methods:**

A cross sectional community based survey was conducted in May and June 2014. A multistage sampling technique was used to randomly select 524 reproductive aged women (15–49 years). Data was collected through interview using a pre-tested and structured questionnaire. Univariate analysis was done to determine the prevalence for Implanon use, to assess general characteristics of respondents, and to produce summaries. Bivariate analysis was conducted to examine the relationship between each independent variable with the dependent variable. Multivariate logistic regression was conducted to identify factors influencing Implanon use by controlling effect of confounding variables.

**Results:**

Of all the women, 444 (84.7%) had heard of Implanon. Health extension workers were the primary source of information on Implanon as mentioned by 376 (71.8%) of the respondents. Little more than seven women in every ten, 319 (71.8%), had good knowledge of Implanon and 248 (55.5%) of the women had supportive attitudes towards Implanon use. Among our sample, 10.1% women were using Implanon, 33 (62.3%) reported having received their Implanon at a health post from health extension worker. Women’s employment (AOR: 2.73, 95% CI: 1.20–6.21), the number of modern contraceptive methods known (AOR: 2.24, 95% CI: 1.09–4.62), and the number of contraceptive methods ever used (AOR: 11.0, 95% CI: 5.06–23.90) were positively associated with Implanon use.

**Conclusion:**

Trained health extension workers played a major role in information and service provision of Implanon. However, this study revealed that a significant number of women had incorrect information regarding Implanon. Hence, health extension workers and other health professionals should provide appropriate counseling and education regarding Implanon and other contraceptives.

## Plain English Summary

Since 2009, the Ethiopian government expanded the provision of Implanon. Implanon is easy to insert, does not require daily use, protects well against pregnancy for 3 years and there is no delay in return to fertility upon removal. Despite these features, its use by rural Ethiopian women has not been well investigated. Thus, this study assessed utilization and factors that affect Implanon use by women in rural areas of northern Ethiopia.

The study interviewed 524 women in two districts of Tigray region, northern Ethiopia in May-June 2014. Out of these women, 10% were using Implanon. Many of the women (62.3%) received Implanon from a health post by health extension workers. Seven in ten women (71.9%) and more than five-in-ten women (55.9%) had good knowledge of Implanon and favorable attitudes towards its use.

Women with paid employment, who knew different contraceptive methods and had history of contraceptive use, had a higher chance of using Implanon.

Health extension workers played a substantial role in improving access to Implanon for rural women in Tigray region, Northern Ethiopia. Nevertheless, the 10% Implanon utilization rate may be increased further if the quality of counseling and education regarding Implanon and other contraceptives methods by health professionals is improved.

## Background

In Ethiopia, there has been an increase in contraceptive use and a decline in the unmet need over the past couple of decades [1]. The contraceptive prevalence rate (CPR) has increased from 15% in 2005 to 29% in 2011 and the unmet need has decreased from 34% in 2005 to 25% in 2011 [1, 2]. A recent mini Ethiopian Demographic Health Survey (EDHS) conducted in 2014, showed the national CPR increased to 40% [3]. However, this increase was not sufficient to meet the national CPR target of 66%, which was set for the year 2015 [4].

Ethiopia’s CPR is highly dependent on short-term contraceptive methods (nearly 21% from injectable contraception), while the use of implants was only 3.4% (12% share from total CPR) [2]. The Federal Ministry of Health (FMOH) of Ethiopia aimed to provide 20% of all family planning (FP) clients with long-acting contraceptive methods by 2015 [4]. To achieve this target, in 2009,  the FMOH introduced community level Implanon insertion by trained health extension workers (HEWs) [5, 6].

Implanon is a single-rod contraceptive implant which provides up to 3 years of protection from pregnancy [7]. This method is easy and convenient with no daily use or frequent follow-up. It provides long-term protection and there is no delay in return to fertility upon removal. Implanon is highly effective, has a very low failure rate, and is safe with rare complications during insertion or removal [8]. Despite these features, its use is low. Among married women between the ages of 15 and 49 around the globe, 53% use a modern method of contraception but less than one percent use implants [8].

The health extension program (HEP) is a community based program introduced in the Ethiopian health system in 2003. At the center of the HEP there are trained HEWs stationed at health posts (HP) [5, 9]. HEWs are full-time salaried employees with a year of professional training, which includes training on the implementation of 16 health packages, one of which is FP. They are from the local community, speak the local language, and are assigned two per HP to serve 5000 individuals. FP services by HEWs include counseling and provision of injectables, pills and condoms [5, 9]. The community based Implanon insertion was later piloted and added as one the roles of HEWs [5, 6]. Before Implanon training to HEWs, Implanon insertion was only provided by nurses or higher level clinical service providers [10].

As part of their routine activities, HEWs train families in their catchment area to become ‘model families’ on the various health packages provided by HEWs. HEWs conduct follow-up visits to identify households that have implemented relevant health packages, and have therefore, met the requirements for graduation and certification as a model family. Model families that fulfill the requirements are recommended for graduation and certification to their respective district health office [11, 12]. This is an approach of giving recognition to families who implemented health packages so that neighboring families are encouraged to do the same. To strengthen and support the work of HEWs at the community level, in 2010/2011 the Ethiopian FMOH introduced a new community mobilization structure called women development groups (WDGs). This is a group of 25 to 30 neighboring households. A development group is further divided into five Health Development Armies (HDA). In other words, six women in a neighborhood form a HDA where one of the six women, with better knowledge on health and health related issues, becomes the leader of the army [13].

Given that Implanon insertion by HEWs is a new community based initiative in Ethiopia, there is no enough evidence on its prevalence or factors associated with its use. Thus, this study aimed at assessing the use of Implanon and factors associated with its use among reproductive aged women in rural areas of Tigray Region, Ethiopia. This study was conducted 5 years after the community based Implanon provision was introduced.

## Methods

### Study area and population

A community-based cross-sectional study was conducted in Saesie-Tsaeda Emba and Ofla districts of Tigray Region, Ethiopia from May to June 2014. The total population of the Saesie-Tsaeda Emba district was 169,008, while it was 144,220 in Ofla district [14].

Reproductive aged women (15–49 years) residing in rural areas of the study districts were considered eligible for inclusion. Women who were critically ill during data collection were excluded. To determine the sample size, a single population proportion formula was used. In calculating the sampling size, we assumed a proportion of implants in Tigray region (0.056%) [2], a confidence level (CI) of 95%, a 3% degree of precision, design effect of 2 and a 10% non-response rate. With these assumptions and the help of EPI INFO statcal, the total estimated sample size was 524.

### Sampling technique

A multistage sampling technique was used to select the study subjects. The study districts were selected purposefully as community-based Implanon provision was first piloted in these districts. There were 48 Kebeles (villages) in the two districts, seven from Saesie-Tsaeda Emba and six from Ofla were randomly selected for the study. Households with eligible reproductive aged women were selected using a random-walk with random start method, and an eligible woman in a selected household was interviewed. The calculated sample size was allocated proportionally based on the number of eligible women in each district. For the random walk, first the center of a randomly selected sub-kebele was identified and spinning a pen chose the direction to start the survey. From the central direction, the first five households in that direction were approached; the first household was selected by drawing a number between one and five. From that household, interviewers followed the road or path, turning to the next road or path in serpentine fashion to visit every fifth household with eligible woman for interview until the predetermined number of households (40) was reached. In a household when no eligible woman was found, the next household was taken, and if two or more eligible women were found in same household, one woman was randomly selected to avoid intra-class correlation.

### Data collection

A structured pre-tested questionnaire was used for all interviews. The questionnaire was prepared in English, translated into the local language (Tigrigna) and back translated to check for consistency. Information collected included socio-demographic and economic characteristics, reproductive history, knowledge, attitude and utilization of Implanon. The questionnaire was adapted from EDHS considering the local context [2]. Thirteen diploma nurses, who can speak the local language, were recruited as data collectors while two degree holder nurses were employed as supervisors. Training was given to the data collectors and supervisors for two consecutive days on the objectives and survey methodology, techniques of interviewing and questionnaire handling, data quality, ethical issues, and on their roles and responsibilities. The questionnaire was pretested on 26 women in an area with similar characteristics.

Data quality was ensured through training, supervision and pretesting. There was daily supervision of data collection. Completed questionnaires were checked for completeness and consistency on the spot by supervisors.

### Measurement

The measurements of the variables in this study, in particular knowledge and attitudes, were adapted from a previous similar study conducted in a similar setting [15].

The mean score was used to categorize women’s knowledge of Implanon; women who scored above the mean were considered to have good knowledge and women who scored below the mean were considered to have poor knowledge. The same approach was employed to categorize women’s attitude towards Implanon use.

Information regarding economic status, involvement in women’s development group and being a model family were collected and analyzed based on self-reporting.

### Data analysis

Data were entered and analyzed using statistical packages for social sciences (SPSS) version 20 for windows (SPSS Inc, Chicago, IL, USA). The predictors of Implanon utilization were assessed using multiple variable logistic regression analysis. The effect size of predictors was estimated using adjusted odds ratio (OR) with 95% CI. A *p*-value of less than 0.05 was considered as statistically significant for all tests. Hosmer-Lemeshow and multi - collinearity tests were done to assess the goodness-of-fit of the model and the presence of collinearity of the variance, respectively.

## Results

### Socio-demographic characteristics

In this study, all women were 15–49 years, with a mean age of 31 years. Predominantly, participants reported their religion as Orthodox (521; 99.4%) and as belonging to the Tigrawot ethnic group (522; 99.6%). In regards to education, 311 (59.4%) had no education, 127 (24.2%) had primary education, and 86 (16.4%) had secondary education or above. Three hundred and one women (57.4%) were married, 74 (14.11%) were single, 122 (23.3%) were divorced and 27 (5.2%) were widowed. Close to one-third of participants (159; 30.3%) were not employed, farming was the most common occupation as mentioned by 236 (45%) (Table 1).

**Table 1:** Socio-demographic characteristics of women in Saesie-Tsaeda Emba and Ofla districts, Northern Ethiopia, May-June, 2014 (*n* = 524)

Variable	*N*(%)
Age	
15–19 20–24 25–29 30–34 35–39 40–44 45–49	51(9.9) 80(15.6) 101(19.6) 91(17.7) 99(19.3) 56(10.9) 36(7.0)
Educational status	
No education Primary Secondary and higher	311(59.4) 127(24.2) 86(16.4)
Marital status	
Single Married Divorced Widowed	74(14.1) 301(57.4) 122(23.3) 27(5.2)
Occupation	
Farmer Housewife/Student/Unemployed Petty trade^a^ Daily labor^b^ Government/private employee	236(45.0) 159(30.3) 70(13.4) 37(7.1) 22(4.2)
Perception on own economic status	
Very poor Poor Average Well to do	12(2.3) 86(16.4) 403(76.9) 23(4.4)
Religion	
Orthodox Christian Muslim	521(99.4) 3(0.6)
Ethnicity	
Tigrawot Other^c^	522(99.6) 2(0.4)
Participation in women development groups	
No participation Member Development leader	134(25.6) 331(63.2) 59(11.3)
Women’s family graduation as model family	
Yes No	322(61.4) 202(38.6)

^a^Petty trade: informal trade and of low profit, ^b^Daily labor = a labor work paid on daily basis, ^c^(Amhara (1), Agewu (1))

### Reproductive characteristics

Among the women, 439 (83.8%) had ever been pregnant, and the number of pregnancies ranged from 1 to 10 with average of 4.  Of all the women, 56 (10.7%) reported ever having an abortion. With regard to birth, 436 (83.2%) of women had ever given birth, the number of living children ranged from 1 to 9 with average of 3.

### Information about Implanon and other contraceptive methods

Five hundred and two (95.8%) participants had heard of at least one contraceptive method, and on average women knew five methods. Four hundred and forty-four participants (84.7%) had heard of Implanon. HEWs were reported as the dominant source of information regarding Implanon, as mentioned by 376 (71.8%) participants followed by health professionals (278; 53.1%), and WDG members (210; 40.1%) (Table 2).

**Table 2:** Information of contraceptive methods of reproductive aged women in Saesie-Tsaeda Emba and Ofla districts, Northern Ethiopia 2014 (*n* = 524)

Variable	*n* (%)
Information of any contraceptive and Implanon	
Heard of any contraceptive Heard of modern contraceptive Heard of Implanon	502(95.8) 502(95.8) 444(84.7)
Source of information on Implanon^a^	
Health extension workers Health professionals Women development group Family/friend/neighbor Radio/television Community conservation Print materials	376(71.8) 278(53.1) 210(40.1) 142(27.1) 141(26.9) 70(13.4) 47(10.0)
Places women can obtain Implanon^a^	
Health post Health center Hospital Private for-profit clinic Non-governmental clinic	391(74.6) 372(71.0) 198(37.8) 37(7.1) 2(0.4)
Knowledge on payment for Implanon (*n* = 440)	
Implanon was free of charge	408(92.7)
Discussion with and support of husbands (*n* = 301)	
Discussed Implanon use with husband Husband support Implanon use	190(63.1) 181(60.1)

^a^Multiple responses were possible

Women were asked where they could access Implanon, HPs and HCs followed by hospital were the three widely cited sources as mentioned by 391 (74.6%), 372 (71.0%), and 198 (37.8%) respectively. Among the women who had responded on payment for Implanon, 408 (92.7%) of them stated that Implanon was free. Of the 301 married participants, 190 (63.1%) had discussed Implanon with their husband, and 181 (60.1%) reported support from their husband for Implanon use (Table 2).

### Knowledge of facts about Implanon

Overall, seven in ten women, 319 (71.8%) had good knowledge of Implanon. Four hundred twenty-five (95.7%) women knew that Implanon can provide effective protection from pregnancy for up to 3 years, and 372 (83.8%) knew that it requires a minor surgical procedure for removal (Table 3).

**Table 3:** Knowledge of Implanon by reproductive aged women in Saesie-Tsaeda Emba and Ofla districts, Northern Ethiopia 2014 (*n* = 444)

Items	Yes	No
	*N* (%)	*N* (%)
Implanon can provide effective protection from pregnancies for up to 3 years	425(95.7)	19 (4.3)
Implanon requires a minor surgical procedure for removal	372(83.8)	72 (16.2)
Implanon has no interference with sexual intercourse or desire	328(73.9)	116(26.1)
Implanon can be discontinued at any time	340(76.5)	104(23.5)
Implanon requires little attention after insertion	301(67.8)	143(32.2)

### Attitude towards Implanon use

More than half of the women, 248 (55.9%) had favorable attitudes towards Implanon use. More specifically, 332 (74.8%) did not perceive Implanon to be difficult to remove, and 282 (63.5%) women perceived that Implanon does not have severe side effects. However, a significant number of women had unsupportive attitudes towards Implanon. Ninety-nine (22.3%) women stated that Implanon causes severe changes in bleeding pattern, 89 (20.2%) agreed that insertion and removal is highly painful, and 98 (22.1%) agreed it can restrict normal activities. Also, 63 (14.2%) and 36 (8.1%) women agreed that Implanon has severe side effects and it is difficult to remove, respectively (Table 4).

**Table 4:** Attitude towards Implanon use of reproductive aged women Saesie-Tsaeda Emba and Ofla districts, Northern Ethiopia 2014 (*n* = 444)

Items	Disagree *N* (%)	Not sure *N* (%)	Agree *N* (%)
Implanon causes severe changes in menstrual bleeding pattern	219(49.3)	126(28.4)	99(22.3)
Using Implanon restricts normal activities	261(58.8)	85(19.1)	98(22.1)
Implanon insertion and removal is highly painful	233(52.5)	122(27.5)	89(20.0)
Implanon has severe side effects	282(63.5)	99(22.3)	63(14.2)
It is difficult to remove Implanon	332(74.8)	76(17.1)	36(8.1)

### Utilization of Implanon and other contraceptive methods

Overall, 351 (67.0%) women had used contraceptives. Of these, 85 (16.2%) women had used Implanon (Table 5). The overall prevalence for current contraceptive use was 42.6% and 10.1% for Implanon. Implanon was the second most commonly used method after the contraceptive injection (30.2%). Each method of the emergency contraceptive pill (ECP), oral contraceptive pill, female sterilization, and lactational amenorrhea method (LAM) was used by less than one percent of women.

**Table 5:** Current use of Implanon and other contraceptive methods and related behavior of reproductive aged women in Saesie-Tsaeda Emba and Ofla woredas, Northern Ethiopia 2014 (*n* = 524)

Variable	*n* (%)
Ever used Implanon and other contraceptives	
Ever used any contraceptive Ever used Implanon	351(67.0) 85(16.2)
Reasons for Implanon use (*n* = 85)^a^	
Reversible Effective and long-term protection Easy and convenient to use Fewer side effects Easy to get	84(98.8) 80(94.1) 31(36.5) 16(18.2) 12(14.1)
Current use of Implanon and other contraceptives	
Any contraceptive method Implanon	223(42.6) 53(10.1)
Current use of contraceptives by method (*n* = 223)	
Injectable Implanon Emergency contraceptive pill Pills Female sterilization Lactational amenorrhea method Periodic abstinence	158(30.2) 53(10.1) 3(0.6) 2(0.4) 1(0.2) 1(0.2) 5(1.0)
Location where received Implanon (*n* = 53)	
Health post Health center Hospital	33(62.3) 16(30.2) 4(7.5)
Choice and satisfaction with Implanon (*n* = 53)	
Implanon was their choice Satisfied with Implanon service received No current complaint with Implanon use	53(100.0) 52(98.1) 48 (9.4)
Patient or user card for Implanon (*n* = 53)	
Yes No	48(90.6) 5(9.4)
Intention for early Implanon removal (*n* = 53)	
Yes No	9(17.0) 44(83.0)
Reasons for not currently using Implanon (*n* = 384)	
Use of other methods Desire for more children Medical reason Fear of side effects Not currently married (unmarried/separated/divorced) Little risk of pregnancy Religion	118(30.7) 98(25.5) 62(16.1) 36(9.4) 36(9.4) 33(8.6) 1(0.3)
Intention to use Implanon in the future (*n* = 431)	
Yes No	280(65.0) 151(35.0)

^a^Multiple response was possible

Among current users of Implanon, 33 (62.3%) had received Implanon insertion at a HP, 16 (30.2%) received it at a HC, and 4 (7.5%) received it at a hospital. All current users of Implanon reported that it was their choice. Fifty-two (98.1%) women were satisfied with the Implanon service they received and 48 (90.4%) had no complaint with current Implanon use.

The three major reasons mentioned by non-users of Implanon for not using the method were use of other methods (118; 30.7%), desire to become pregnant (98; 25.5%), and medical reasons (62; 16.1%). In addition, the fear of side effects and marital status (unmarried/separated/divorced) were mentioned in similar proportions (36; 9.4%).

### Factors that affect utilization of Implanon

On bivariate analysis; age of women, marital status, number of living children, number of contraceptive methods known, number of contractive methods ever used and being a member of a model family were associated with current use of Implanon. However, on multivariate analysis, only women’s employment, number of contraceptive methods known and number of ever used contraceptives were significantly associated with Implanon use. Women who were currently working were more than two times more likely to use Implanon when compared to women who were not currently working (AOR: 2.73, 95% CI: 1.20–6.21) (Table 6).

**Table 6:** Predictors of Implanon use among reproductive age women in Saesie-Tsaeda Emba and Ofla districts, Northern Ethiopia 2014 (*n* = 524)

Characteristics	Current use of Implanon		Odds ratio	
	Yes	No	COR (95% CI)	AOR (95% CI)
Age (*n* = 514)				
15–24	7(5.3)	124(9.7)	1	1
25–29	7(6.9)	94(93.1)	1.23 (0.45, 3.89)	0.63(0.18, 2.14)
30–34	19(20.8)	72(79.1)	4.68 (1.87, 11.66)*	2.18(0.61, 7.83)
35–39	10(10.1)	89(89.9)	1.99 (0.73, 5.43)	1.16(0.26, 5.09)
> =40	10(10.9)	82(89.1)	2.16 (0.79, 5.90)	1.27(0.28, 5.77)
Marital status				
Other^um^	11(4.9)	212(95.1)	1	1
Married	42(14.0)	259(86.0)	3.13 (1.57, 6.22)*	2.14(0.95, 4.83)
Employment				
Not currently working	10(6.3)	149(93.7)	1	1
Currently working	43(11.8)	322(88.2)	1.99 (0.97, 4.07)	2.73(1.20, 6.21)*
Member of model family				
No	12(5.9)	190(94.1)	1	1
Yes	41(12.7)	281(87.3)	2.31 (1.18, 4.51)*	1.60(0.72, 3.57)
Number of living children				
0	3(3.2)	90(96.8)	1	1
1 to 2	16(10.3)	140(89.7)	3.43 (0.97, 12.10)	1.36(0.32, 5.83)
3 to 4	15(9.6)	142(90.4)	3.17 (0.89, 11.26)	1.08(0.20, 5.97)
> 4	19(16.1)	99(83.9)	5.76 (1.65, 20.11)*	2.09(0.34, 12.9)
Number of methods known				
Poor	16(5.8)	261(94.2)	1	1
Good	37(15.0)	210(85.0)	2.87(1.56, 5.31)*	2.24(1.09, 4.62)*
Number of methods ever used				
Poor	9(2.6)	335(97.4)	1	1
Good	44(24.4)	136(75.6)	12.04(5.72, 25.35)*	11.0(5.06, 23.90)*

^um^ = (single, divorced, widowed), * = *p* < 0.05

Women who had good knowledge of contraceptive methods were over two times more likely to use Implanon when compared with women who had poor knowledge of contraceptive methods (AOR: 2.24, 95% CI: 1.09–4.62). Similarly, women with a history of contraceptive use were eleven times more likely to use Implanon when compared with women with no history of contraceptive use (AOR: 11.0, 95% CI: 5.06–23.90).

## Discussion

This study showed more than eight women in every ten (84.7%) had heard of Implanon. HEWs were the primary (71.8%) source of information on Implanon followed by health professionals (53.1%) and WDG members (40.1%). More than seven women in every ten (71.8%) were knowledgeable about Implanon and more than half of the women (55.9%) had supportive attitudes towards Implanon. The overall prevalence of contraceptive use was 42.6% and 10.1% for Implanon. Implanon was the second most commonly used method after injectables (30.2%). Among the current users of Implanon, 62.3% had received Implanon insertion at HP by HEWs. Reasons for non-current use of Implanon mentioned were use of other methods, desire for a child, and medical reasons. Current employment, number of contraceptive methods known and number of contraceptive methods ever used were the significant variables associated with current Implanon use in the multivariate analysis.

In this study 10.1% of the women were using Implanon. This is much higher than the finding of the EDHS 2011 which showed a 2.3% overall utilization of Implants [2]. This study also showed majority of users received it from HPs by HEWs and HEWs were the primary source of information on Implanon. These findings may show the community-based Implanon service provision by HEWs can contribute to the increasing use of Implanon by rural women. Thus, we recommend the Ethiopian government and other developing countries to strengthen such community-based Implanon service provision to meet the demand of underserved rural women for long-acting contraceptive methods.

The main reasons for not using Implanon were the preference of other contraceptive methods, in particular short-acting contraceptive methods, the desire for more children and medical reasons. This is consistent with the findings of other studies in Mekelle, Ethiopia [15, 16]. In addition, it should be noted that a significant number of women had incorrect information on Implanon use. This might show that the quality of counseling and education of contraceptives was poor. Hence, while strengthening and scaling-up community-based Implanon service provision, health extension workers and other health professionals should be trained to give appropriate counseling and education on the benefits and side effects of Implanon and other contraceptive methods. This would be helpful in increasing the number of women who choose Implanon and other long acting contraceptive methods.

A similar study in Ethiopia indicated that model families were more likely to use contraceptives when compared with non-model families [12]. This finding is in line with our study in which, the majority of the Implanon users were women participating in WDG either as a member or a leader and members of the graduated model family. Although it was not significantly associated on the multivariate analysis, the bivariate analysis showed that members of a model family were two times more likely to use Implanon. This may imply that women who had close contacts with HEWs, through the community mobilization structure, had a higher chance of using Implanon and other contraceptives. Hence, such innovative community mobilization structures should be strengthened to improve demand and uptake of contraceptives by rural women in Ethiopia.

Our study showed currently employed women were more than three times more likely to use Implanon when compared to unemployed women (AOR: 3.24, 95% CI: 1.37–7.67). This finding is supported with findings from a study conducted on women’s empowerment as a determinant of contraceptive use in Ethiopia among married women. The findings of this study revealed that the likelihood of contraceptive use was almost 40% greater for women who were currently working [17]. This may imply that women with good economic status may have a higher chance of independent decision-making, financial autonomy, and may more easily receive information from health care providers or media and be able to persuade their husband.

The number of living children was positively associated with women’s use of Implanon. Similarly, a study conducted on the determinants of modern contraceptive in North Shoa Zone, Amhara Region, Ethiopia revealed that the number of living children was significantly associated with the use of contraceptives [18]. This might indicate multiparous women think that the number of children that they already have could be enough for them and do not desire more children. On the other hand, this might show that women with more births would be more likely to be older and they might not be interested to have more children. As a result, they may look for Implanon and other long acting contraceptives to limit their family size.

This study showed that the number of modern contraceptives known was significantly associated with women’s current use of Implanon. This finding is in line to the finding of a study conducted on awareness and determinants of family planning in Jimma, Ethiopia which revealed a significant association between the number of contraceptive methods known and use of contraceptives [19]. Similarly, in this study, the number of methods ever used was significantly associated with current use of Implanon. This finding was supported by a study conducted among married women in Goba town, Bale zone, South East Ethiopia [20]. Another study conducted on knowledge and attitudes towards use of long acting reversible contraceptives among women of reproductive age in Lubaga division, Kampala district, Uganda, revealed similar finding [21]. This may be correlated with the fact that women who have ever used contraceptives may know advantages of using contraceptives, may have adequate education and counseling on contraceptives. Moreover, ever users may shift from short acting to long acting methods or they might switch among the long acting contraceptive methods. Thus, encouraging those who have ever used any contraceptive methods to switch to long acting contraceptives might be helpful in increasing the use of long acting contraceptives.

This study has several limitations. First, the study did not address some factors which may influence Implanon use. These factors include partner’s involvement and distance to health facility. Second, the study districts were selected purposefully and are both rural, thus, may not be representative to urban settings. We recommend further study on Implanon use and associated factors in urban settings of Ethiopia. Third, some of the variables such as contraceptives methods known, knowledge of Implanon, and attitudes towards Implanon were defined operationally taking the mean value as cut off point. Hence, this might limit the interpretation and comparison of the findings as other studies may not use similar definition and measurement of variables.

## Conclusion

This study showed HEWs were the primary source of information on Implanon at the community level. Many women received Implanon insertion at HPs from HEWs. Hence, trained HEWs on Implanon insertion are playing important role in educating, mobilizing, counseling, and providing Implanon insertion.

Although, the large majority of women had heard of Implanon and were knowledgeable on facts of Implanon, more than four women in every ten had unfavorable attitudes towards Implanon use. This suggests that more has to be done to reverse negative attitudes. Improving counseling and education has paramount importance in improving the utilization of Implanon and other contraceptives.

## Abbreviations

AOR: Adjusted odds ratio
CI: Confidence interval
CPR: Contraceptive prevalence rate
ECP: Emergency contraceptive pill
EDHS: Ethiopian Demographic Health Survey
ERC: Ethical Review Committee
FMOH: Federal Ministry of Health
FP: Family planning
HC: Health Center
HDA: Health Development Army
HEP: Health extension program
HEW: Health extension worker
HP: Health post
LAM: Lactational amenorrhea method
OR: Odds ratio
SNNPR: Sothern Nations and Nationalities Peoples Region
SPSS: Statistical packages for social sciences
WDG: Women Development Group
